# Mammographic breast density and risk of breast cancer in women with atypical hyperplasia: an observational cohort study from the Mayo Clinic Benign Breast Disease (BBD) cohort

**DOI:** 10.1186/s12885-017-3082-2

**Published:** 2017-01-31

**Authors:** Robert A. Vierkant, Amy C. Degnim, Derek C. Radisky, Daniel W. Visscher, Ethan P. Heinzen, Ryan D. Frank, Stacey J. Winham, Marlene H. Frost, Christopher G. Scott, Matthew R. Jensen, Karthik Ghosh, Armando Manduca, Kathleen R. Brandt, Dana H. Whaley, Lynn C. Hartmann, Celine M. Vachon

**Affiliations:** 1 Department of Health Sciences Research, Division of Biomedical Statistics and Informatics, Mayo Clinic, Rochester, MN USA; 2 Department of Subspecialty General Surgery, Mayo Clinic, Rochester, MN USA; 3 Department of Cancer Biology, Mayo Clinic, Jacksonville, FL USA; 4 Department of Anatomic Pathology, Mayo Clinic, Rochester, MN USA; 5 Department of Health Sciences Research, Biomedical Statistics and Informatics, Mayo Clinic, Jacksonville, FL USA; 6 Department of Medical Oncology, Division of the Women’s Cancer Program, Mayo Clinic, Rochester, MN USA; 7 Department of General Internal Medicine, Division of the Breast Diagnostic Clinic, Mayo Clinic, Rochester, MN USA; 8 Department of Physiology and Biomedical Engineering, Mayo Clinic, Rochester, MN USA; 9 Department of Radiology, Mayo Clinic, Rochester, MN USA; 10 Department of Medical Oncology, Mayo Clinic, Rochester, MN USA; 11 Department of Health Sciences Research, Division of Epidemiology, Mayo Clinic, 200 First Street SW, Rochester, MN 55905 USA

**Keywords:** Mammographic breast density, Breast cancer risk, Atypical hyperplasia

## Abstract

**Background:**

Atypical hyperplasia (AH) and mammographic breast density (MBD) are established risk factors for breast cancer (BC), but their joint contributions are not well understood. We examine associations of MBD and BC by histologic impression, including AH, in a sub﻿cohort of women from the Mayo Clinic Benign Breast Disease Cohort.

**Methods:**

Women with a diagnosis of BBD and mammogram between 1985 and 2001 were eligible. Histologic impression was assessed via pathology review and coded as non-proliferative disease (NP), proliferative disease without atypia (PDWA) and AH. MBD was assessed clinically using parenchymal pattern (PP) or BI-RADS criteria and categorized as low, moderate or high. Percent density (PD) was also available for a subset of women. BC and clinical information were obtained by questionnaires, medical records and the Mayo Clinic Tumor Registry. Women were followed from date of benign biopsy to BC, death or last contact. Standardized incidence ratios (SIRs) compared the observed number of BCs to expected counts. Cox regression estimated multivariate-adjusted MBD hazard ratios.

**Results:**

Of the 6271 women included in the study, 1132 (18.0%) had low MBD, 2921 (46.6%) had moderate MBD, and 2218 (35.4%) had high MBD. A total of 3532 women (56.3%) had NP, 2269 (36.2%) had PDWA and 470 (7.5%) had AH. Over a median follow-up of 14.3 years, 528 BCs were observed. The association of MBD and BC risk differed by histologic impression (p-interaction = 0.03), such that there was a strong MBD and BC association among NP (*p* < 0.001) but non-significant associations for PDWA (*p* = 0.27) and AH (*p* = 0.96). MBD and BC associations for AH women were not significant within subsets defined by type of MBD measure (PP vs. BI-RADS), age at biopsy, number of foci of AH, type of AH (lobular vs. ductal) and body mass index, and after adjustment for potential confounding variables. Women with atypia who also had high PD (>50%) demonstrated marginal evidence of increased BC risk (SIR 4.98), but results were not statistically significant.

**Conclusion:**

We found no evidence of an association between MBD and subsequent BC risk in women with AH.

**Electronic supplementary material:**

The online version of this article (doi:10.1186/s12885-017-3082-2) contains supplementary material, which is available to authorized users.

## Background

Breast biopsies are commonly performed to investigate BC in women with suspicious mammographic or palpable findings, and the majority of them reveal only benign breast lesions. In fact, of the estimated 1.6 million breast biopsies performed in the United States each year [1], approximately 80% are found to be benign [2]. The histologic features of these benign breast disease (BBD) findings are quite varied and can be used to stratify women into groups with significantly different risks of developing a later BC [3, 4]. Atypical hyperplasia (AH) is a high-risk benign lesion found in approximately 10% of benign biopsies [5] and is composed of two histologic subtypes: atypical ductal hyperplasia (ADH) and atypical lobular hyperplasia (ALH). We and others have previously reported that women with AH are at an approximately four-fold risk of subsequent BC [3, 4, 6, 7], and have an approximate 30% cumulative risk at 25 years post biopsy [8]. This long-term risk is similar for women with ADH and those with ALH [6, 8].

In a recent review article we suggested that clinicians consider the use of screening MRIs and pharmacologic agents such as aromatase inhibitors (AIs) and selective estrogen receptor modulators (SERMs) as potential preventive options for women with AH [9]. However, we also recognize that many women diagnosed with AH will never progress to BC. Clinical prevention measures can be costly, and pharmacological agents can induce adverse side effects. Thus, it is important to identify risk factors among women with AH that further stratify BC risk in order to target screening and prevention efforts to those with the highest risk.

Mammographic breast density (MBD), which represents the proportion of tissues that appear white or dense on a mammogram, is a well-established risk factor for breast cancer [10–12]. Women with high MBD have a 3–5 fold increased risk of BC relative to those with low density [13, 14]. It has also been shown that AH is associated with increased MBD [15]. However, to date there have been very few studies examining the association of MBD with BC risk in women with AH, with inconsistent findings. Byrne et al. found no association between percent density and risk in women with AH [16]. Conversely, two other studies have reported increased risk in women with AH who have high MBD [17, 18], although small sample sizes limit the significance of the associations. We previously reported no association between MBD [measured by Wolfe’s parenchymal pattern (PP)] and BC risk in a group of 147 women with AH [19]. Here, we present results in an expanded cohort of 470 women diagnosed with AH between 1985 and 2001 to examine if MBD can further stratify BC risk in women with AH.

## Methods

### Study setting and population

The Mayo Clinic Benign Breast Disease study has been described previously [3] and currently comprises 13,527 women ages 18 to 85 who underwent a benign breast biopsy between 1967 and 2001 at Mayo Clinic in Rochester, MN. Detailed demographic and clinical features and risk factors were identified from medical records and questionnaires [3]. BC events were ascertained from study questionnaires, tumor registry, and review of medical records. The study protocol, including patient contact and follow-up methods, was approved by the Mayo Clinic Institutional Review Board. We excluded all women who refused to allow use of their medical record for research. All women in the BBD cohort with a biopsy between 1985 and 2001 and for whom MBD was available from clinical records﻿,were included in this particular study.

### Histologic examination

The study breast pathologist (DWV) performed histologic review of archived hematoxylin-and-eosin (H&E) slides from the benign biopsies. Histology was classified according to the criteria of Page et al. [4, 7] into the following categories: nonproliferative disease (NP), proliferative disease without atypia (PDWA), and AH. The degree of lobular involution (LI) for each individual was categorized as described previously [20].

### Assessment of mammographic breast density

**Fig. 1:**
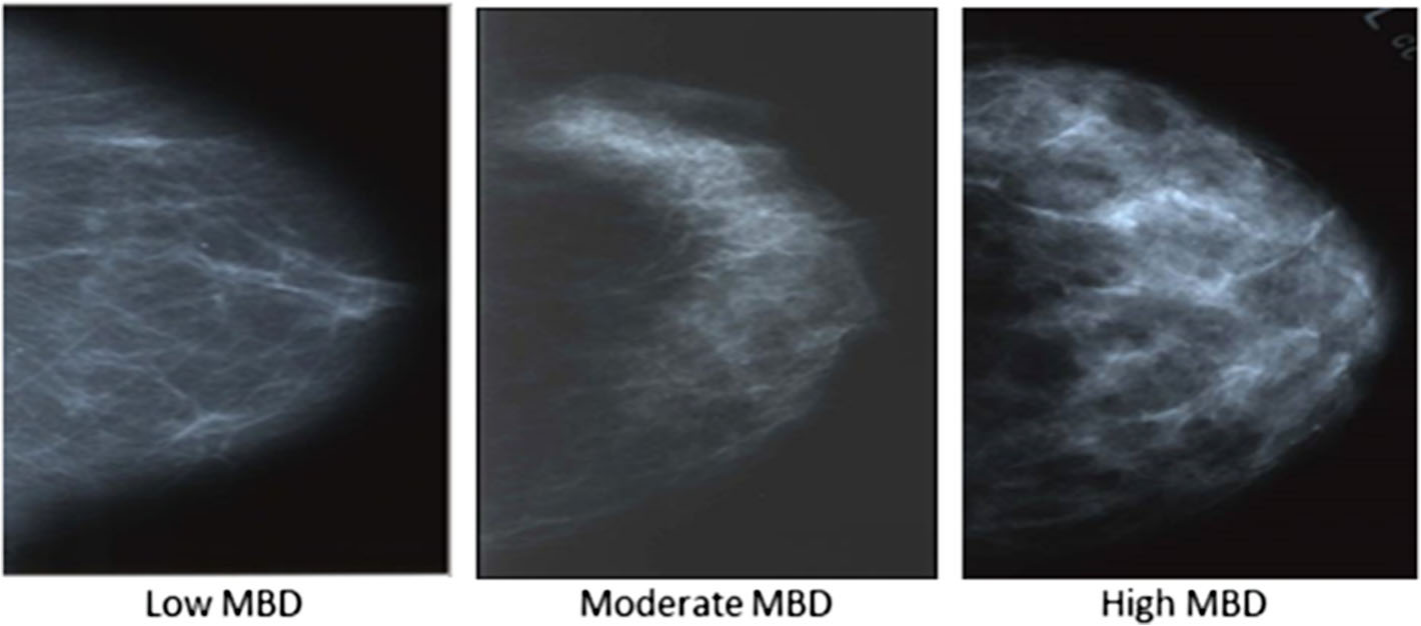
Pattern of mammographic density and corresponding sample sizes. Categories of mammographic density based on parenchymal pattern (PP) and BI-RADS density. Panels from left to right display representative examples of low MBD (PP category N1 [*N* = 60] and BI-RADS category “fatty” [*N* = 9]; moderate MBD (PP categories P1 [*N* = 32] or P2 [*N* = 59], and BI-RADS categories “scattered” [*N* = 55]or “heterogeneously dense” [*N* = 85]); and high MBD (PP category DY [*N* = 131] and BI-RADS category “extremely dense” [*N* = 39])

MBD was available from medical records starting in 1985. From 1985 to June 1996, MBD was measured at Mayo Clinic using Wolfe’s four-category parenchymal pattern (PP) criteria [21]: N1—non-dense, no ducts visible; P1—ductal prominence occupying less than a fourth of the breast; P2—prominent ductal pattern occupying more than a fourth of the breast; and DY—homogenous, plaque-like areas of extreme density [21]. From July 1996 to 2001 MBD was measured using the four density categories of the American College of Radiology Breast Imaging Reporting and Data System (BI-RADS) [22]: almost entirely fat (low density); scattered fibroglandular densities (average density); heterogeneously dense (high density); extremely dense (very high density). For the primary analyses, the density measures above were categorized as low, moderate or high MBD by combining the middle two categories for each (Fig. 1).

Retrieval of mammogram films was attempted on all women with AH over this period. Clinical practice generally saved mammogram films for a ten year period. All available mammographic films were digitized using an Array 2905 laser digitizer (Array Corporation, Netherlands) that has 50 micrometer (limiting) pixel spacing with 12-bit gray scale bit depth. A single expert reader, blinded to BC status, calculated mammographic percent density using the craniocaudal view of the noncancerous breast of women who progressed to breast cancer and the left breast of unaffected women. Percent mammographic density, defined as dense area divided by total area x 100%, was calculated using Cumulus, a computer-assisted thresholding program [23]. Five percent of images were repeated to assess reliability, with a resulting intraclass correlation exceeding 0.93. For the purposes of this study, percent density was classified into four categories: 0-10%, 11-25%, 26-50%, > 50%.

### Statistical methods

Data were summarized using frequencies and percents for categorical variables, and medians and ranges for continuous variables. Associations of MBD with demographic and clinical variables were first assessed using chi-square tests of significance. All variables that were univariately statistically significant were then included in a multivariate logistic regression model to assess the independent effects of these characteristics.

To reduce the possibility of including women with subclinical BC at benign biopsy, women did not contribute person years of observation until six months post-biopsy. Duration of follow-up was calculated as the number of days from that date to the date of BC diagnosis, death, or last contact. In addition, women with prophylactic mastectomies or a diagnosis of lobular carcinoma in situ (LCIS) were censored at the date of such occurrence. We estimated relative risks (RR) using standardized incidence ratios (SIRs) and corresponding 95% confidence intervals (CI), dividing the observed numbers of incident BCs by the population-based expected counts. We calculated expected counts by apportioning each woman’s follow-up into 5-year age groups and multiple calendar periods, thereby accounting for differences associated with these variables. We used the Iowa Surveillance, Epidemiology, and End Results (SEER) registry as the reference population because of its demographic similarities to the Mayo population (80% of cohort members reside in the Upper Midwest). SIRs were calculated both overall and within subgroups defined by histologic, clinical and demographic characteristics. We assessed potential heterogeneity in SIRs across subgroups using Poisson regression analysis, with the log transformed expected event rate for each individual modeled as the offset term.

Cox proportional hazards regression analysis was used to estimate intra-cohort MBD hazard ratios after adjustment for demographic and clinical variables. Statistical tests were two-sided, and analyses were conducted with use of SAS statistical software version 9.4 (SAS Institute Inc., Cary NC). A *p*-value < 0.05 was treated as significant.

## Results

Of the 7999 women in the BBD cohort diagnosed between 1985 and 2001, 6271 (78.4%) had MBD data within one year prior to biopsy (3532 with NP, 2269 with PDWA and 470 with AH). A summary of the number of women by levels of histologic impression, MBD, BMI and breast cancer status can be found in Additional file 1. Older women were more likely to have MBD values than younger women. MBD data availability did not differ significantly across year of biopsy, number of atypical foci, type of atypia (ADH vs. ALH), extent of lobular involution or body mass index, (*p*-value > 0.05 for each, data not shown).

We observed an association between histologic category of BBD and MBD, in that women with NP were more likely to fall into the low MBD category (699/3532, 19.8%) than those with PDWA (364/2269, 16.0%) or AH (69/470, 14.7%, chi-square *p*-value < 0.001). After accounting for age at biopsy and BMI, results were even more striking: women with AH were more than twice as likely to be in the high MBD category vs. the low category than those with NP (logistic regression odds ratio 2.10, 95% CI 1.51-2.93).

**Fig. 2:**
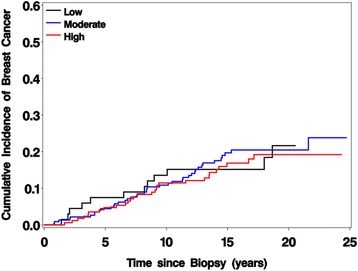
Cumulative breast cancer incidence by extent of mammographic breast density in women with atypical hyperplasia. Curves account for death as a competing event

Over a median follow-up of 14.3 years for the 6271 women, 528 BCs were observed (224 in women with NP, 222 in women with PDWA and 82 in women with AH). We observed a strong positive dose–response association between MBD and BC risk in women with NP (test for heterogeneity in SIRs *p* < 0.001), and a modest but non-significant association in women with PDWA (*p* = 0.27, Table 1). In contrast, risk of breast cancer did not appreciably differ across density categories for women with AH (SIR for low density 3.40, for moderate density 3.48, and for high density 3.25, test for heterogeneity *p*-value = 0.96, Table 2). BC cumulative incidence curves also overlapped considerably across the three levels of extent of MBD for these women (Fig. 2). Tests for interaction between histologic impression (modeled as a categorical variable) and MBD (modeled as an ordinal variable) revealed that histologic impression significantly modified the association between MBD and breast cancer risk (*p* = 0.03). Because the null finding in AH differed from what we had seen in the other two histologies, we examined the subset of women with AH more closely. Of the 470 eligible women with AH, 69 (15%) had low, 231 (49%) had moderate, and 170 (36%) had high extent of MBD, respectively. Associations of MBD with demographic and clinical characteristics in women with AH are provided in Table 2. Univariate results showed several associations with MBD. After multivariate adjustment, age at biopsy (*p* = 0.001), type of MBD measurement (*p* < 0.001), degree of lobular involution (*p* = 0.03), and BMI (*p* < 0.001) remained statistically significant. Compared to women with high MBD values, those with low values tended to be older, to have a higher BMI, and to have more extensive LI. In addition, women with high or low MBD were more likely to have had a PP density measurement.

**Table 1:** Associations of extent of mammographic breast density with breast cancer risk by levels of benign histologic impression

	Low Density				Medium Density				High Density				*p*-value^a^
Characteristic	N	Obs	Exp	SIR (95% CI)	N	Obs	Exp	SIR (95% CI)	N	Obs	Exp	SIR (95% CI)	
Histologic Impression													
NP	699	30	40.07	0.75 (0.50, 1.07)	1586	99	80.27	1.23 (1.00, 1.50)	1247	95	56.69	1.68 (1.36, 2.05)	<0.001
PDWA	364	31	22.15	1.40 (0.95, 1.99)	1104	113	58.76	1.92 (1.58, 2.31)	801	78	43.50	1.79 (1.42, 2.24)	0.27
AH	69	12	3.53	3.40 (1.76, 5.93)	231	41	11.77	3.48 (2.50, 4.73)	170	29	8.92	3.25 (2.18, 4.67)	0.96

Standardized incidence ratios and corresponding 95% confidence intervals, comparing the observed number of breast cancer events to those expected based on incidence rates from Iowa SEER data. Analyses account for the effects of age and calendar period

*NP* non-proliferative disease, *PDWA* proliferative disease without atypia, *AH* atypical hyperplasia, *N* number of individuals, *Obs* observed number of breast cancer events, *Exp* expected number of breast cancer events, *SIR* standardized incidence ratio, *CI* confidence interval

^a^ *P*-value, test of heterogeneity in SIRs across columns

**Table 2:** Associations of mammographic breast density with demographic and clinical variables

Characteristic	Low (*N* = 69, 15%)	Moderate (*N* = 231, 49%)	High (*N* = 170, 36%)	Total (*N* = 470)	*p*-value^a^	Multivariate *p*-value^b^
Age at Benign Biopsy					<0.001	0.001
< 45	7 (10.1%)	15 (6.5%)	32 (18.8%)	54 (11.5%)		
45-55	9 (13.0%)	76 (32.9%)	67 (39.4%)	152 (32.3%)		
55+	53 (76.8%)	140 (60.6%)	71 (41.8%)	264 (56.2%)		
Type of Density Measure					<0.001	<0.001
BI-RADS	9 (13.0%)	140 (60.6%)	39 (22.9%)	188 (40.0%)		
PPAT	60 (87.0%)	91 (39.4%)	131 (77.1%)	282 (60.0%)		
Number of Atypical Foci					0.31	
1	47 (68.1%)	126 (54.5%)	96 (56.5%)	269 (57.2%)		
2	15 (21.7%)	61 (26.4%)	42 (24.7%)	118 (25.1%)		
3+	7 (10.1%)	44 (19.0%)	32 (18.8%)	83 (17.7%)		
Type of Atypia					0.004	0.11
ADH	41 (59.4%)	116 (50.2%)	65 (38.2%)	222 (47.2%)		
ALH	27 (39.1%)	96 (41.6%)	96 (56.5%)	219 (46.6%)		
ADH and ALH	1 (1.4%)	19 (8.2%)	9 (5.3%)	29 (6.2%)		
Involution					<0.001	0.03
Missing	2	11	7	20		
None	1 (1.5%)	19 (8.6%)	26 (16.0%)	46 (10.2%)		
Partial	41 (61.2%)	124 (56.4%)	112 (68.7%)	277 (61.6%)		
Complete	25 (37.3%)	77 (35.0%)	25 (15.3%)	127 (28.2%)		
BMI					<0.001	<0.001
Missing	1	2	2	5		
< 25	25 (36.8%)	78 (34.1%)	101 (60.1%)	204 (43.9%)		
25-29	19 (27.9%)	70 (30.6%)	35 (20.8%)	124 (26.7%)		
30+	24 (35.3%)	81 (35.4%)	32 (19.0%)	137 (29.5%)		

Values presented as number (percent)

^a^ Chi-square tests

^b^ Multicategorical nominal logistic regression analysis modeling extent of density as the outcome variable. Model includes all variables found to be univariately significant (*p* < 0.05)

Comparisons of clinical and demographic characteristics by type of density measure (BIRADS versus PP) in women with AH revealed very few differences (Additional file 2). Women with BI-RADS density values were slightly more likely to have been diagnosed with ADH (either alone or in combination with ALH) than those with PP values (60.6% vs. 48.6%). No other attributes differed across MBD measurement type, supporting our decision to combine the two MBD measurement types.

We also examined associations between MBD and breast cancer risk within subsets of women with AH. We found no evidence of heterogeneity in risk by MBD when examining subsets defined by type of MBD measure (PP vs. BI-RADS), age at benign biopsy, number of atypical foci, type of AH, or BMI, although sample sizes in some of these subsets were small (Table 3).

**Table 3:** Associations of extent of mammographic breast density with breast cancer risk in women with atypical hyperplasia

	Low Density				Medium Density				High Density				*p*-value^a^
Characteristic	N	Obs	Exp	SIR (95% CI)	N	Obs	Exp	SIR (95% CI)	N	Obs	Exp	SIR (95% CI)	
Overall	69	12	3.53	3.40 (1.76, 5.93)	231	41	11.77	3.48 (2.50, 4.73)	170	29	8.92	3.25 (2.18, 4.67)	0.96
Type of Density Measure													
PPAT	60	11	3.16	3.48 (1.74, 6.23)	91	17	5.39	3.15 (1.84, 5.05)	131	24	7.33	3.28 (2.10, 4.87)	0.97
BIRADS	9	1	0.37	2.73 (0.07, 15.13)	140	24	6.37	3.77 (2.41, 5.60)	39	5	1.59	3.14 (1.02, 7.31)	0.89
Age at Biopsy													
< 45	7	2	0.30	6.61 (0.80, 23.79)	15	2	0.32	6.17 (0.75, 22.21)	32	4	1.20	3.33 (0.91, 8.53)	0.66
45-55	9	0	0.39	NA	76	15	3.64	4.12 (2.31, 6.80)	67	14	3.65	3.84 (2.10, 6.44)	0.21
55+	53	10	2.84	3.53 (1.69, 6.49)	140	24	7.80	3.08 (1.97, 4.58)	71	11	4.07	2.70 (1.35, 4.84)	0.83
Number of Atypical Foci													
1	47	6	2.73	2.20 (0.81, 4.79)	126	19	6.80	2.79 (1.68, 4.36)	96	16	4.76	3.36 (1.92, 5.46)	0.65
2	15	4	0.52	7.73 (2.11, 19.81)	61	14	2.81	4.98 (2.72, 8.36)	42	6	2.42	2.48 (0.91, 5.40)	0.16
3+	7	2	0.29	6.98 (0.84, 25.13)	44	8	2.15	3.72 (1.61, 7.32)	32	7	1.74	4.03 (1.62, 8.29)	0.76
Type of Atypia													
ADH	41	7	2.18	3.21 (1.29, 6.61)	116	17	5.74	2.96 (1.73, 4.75)	65	15	3.48	4.31 (2.41, 7.11)	0.57
ALH	27	5	1.28	3.92 (1.27, 9.12)	96	20	5.14	3.89 (2.38, 6.01)	96	13	4.92	2.64 (1.40, 4.52)	0.51
ADH and ALH	1	0	0.08	NA	19	4	0.89	4.48 (1.22, 11.48)	9	1	0.51	1.94 (0.05, 10.79)	0.56
BMI at Biopsy													
< 25	25	2	1.45	1.38 (0.17, 4.97)	78	18	3.96	4.55 (2.69, 7.19)	101	17	5.17	3.29 (1.91, 5.27)	0.16
25-29	19	5	1.01	4.96 (1.61, 11.55)	70	10	3.37	2.97 (1.42, 5.46)	35	4	1.95	2.05 (0.56, 5.25)	0.42
30+	24	5	1.07	4.66 (1.51, 10.86)	81	13	4.39	2.96 (1.57, 5.07)	32	7	1.76	3.97 (1.60, 8.17)	0.65

Standardized incidence ratios and corresponding 95% confidence intervals, comparing the observed number of breast cancer events to those expected based on incidence rates from Iowa SEER data. Analyses account for the effects of age and calendar period

*N* number of individuals, *Obs* observed number of breast cancer events, *Exp* expected number of breast cancer events, *SIR* standardized incidence ratio, *CI* confidence interval

^a^ *P*-value, test of heterogeneity in SIRs across columns

Due to concerns that both the PP and BI-RADS MBD measures are subjective, we conducted a series of sensitivity analyses in a group of 212 women (with 32 resulting BC events) for whom mammographic percent density (PD) was available. Results are provided in Table 4. Risk of breast cancer did not appreciably differ across the lower three PD categories (SIR 2.54 for 0-10%, 3.75 for 11-25%, and 2.94 for 26-50%). We observed an SIR of 4.98 (95% CI 0.60-17.92) for women with >50% PD, but this category included only 8 subjects and 2 observed breast cancer events, resulting in a very imprecise point estimate. As with the primary analyses, the test for heterogeneity in the SIRs was non-significant (*p* = 0.76)

**Table 4:** Associations of percent mammographic breast density (PD) with breast cancer risk in a subgroup of women with atypical hyperplasia

Characteristic	No. Women	Person Years	Observed Events	Expected Events	SIR (95% CI)	*p*-value^a^
Overall	212	2469	32	10.15	3.15 (2.16, 4.45)	
Percent Density						0.76
0-10%	59	688	8	3.15	2.54 (1.10, 5.00)	
11-25%	69	777	12	3.20	3.75 (1.94, 6.55)	
26-50%	76	900	10	3.41	2.94 (1.41, 5.40)	
51 + %	8	104	2	0.40	4.98 (0.60, 17.92)	

Standardized incidence ratios and corresponding 95% confidence intervals, comparing the observed number of breast cancer events to those expected based on incidence rates from Iowa SEER data

Analyses account for the effects of age and calendar period

^a^ *P*-value, test of heterogeneity in SIRs

Primary analyses combined the middle two categories of the PP and BI-RADs MBD measures, but secondarily we examined associations with BC risk within each of the four categories. Results were similar for PP P1 (SIR 3.62, CI 1.46-7.45) and P2 (SIR 2.89, CI 1.39-5.32), and for scattered (SIR 3.49, CI 1.60-6.64) and heterogeneously dense BI-RADS density categories (SIR 3.95, CI 2.21-6.51, Additional file 3). Sensitivity analyses retaining the original four-level density values and testing for trend across these values also yielded null results (*p* = 0.83).

Due to concerns that associations of MBD with BC risk may differ depending on time since initial biopsy, we ran sensitivity analyses subsetting to the first 10 years of post-biopsy follow-up. Findings were similar to our overall results: SIR 4.11 (95% CI 1.97-7.56) for low MBD, 3.27 (2.14-4.80) for moderate MBD, and 3.63 (2.18-5.67) for high MBD respectively (test for heterogeneity *p* = 0.82). Also, because analysis of BC risk using SIRs does not allow for formal adjustment of certain potential confounding variables, we re-examined MBD risk associations using intra-cohort Cox proportional hazards regression analyses (Additional file 4). We again found no evidence of association after adjustment for age at biopsy, BMI, type of MBD measure (when applicable) and extent of involution (*p* = 0.69 using the PP/BI-RADS density measure and *p* = 0.47 using the PD measure). Further analyses modeling PD as a one degree-of-freedom linear term, first using the original PD values (*p* = 0.57) and then using square-root-transformed values (*p* = 0.58) yielded similar results.

Finally, we limited events to only the 65 invasive breast cancers, censoring women with DCIS at date of diagnosis. Although SIRs did order in the hypothesized direction (SIRs = 2.62 for low, 3.09 for moderate, and 3.45 for high MBD respectively), relative effect sizes were small and did not approach statistical significance (test for heterogeneity *p* = 0.78). We found no association of MBD with invasive breast cancer using Cox regression analyses (HRs = 1.08 and 1.08 for moderate and high MBD relative to low MBD, *p* = 0.98).

## Discussion

We found the MBD and breast cancer association differed by histologic impression. In particular, there was a strong association among women with NP and a suggestive association among PDWA. However, in our cohort of 470 women diagnosed with AH, we found no convincing evidence of an association between mammographic breast density and subsequent risk of BC. Null associations persisted within most of the AH subsets and after adjustment for relevant demographic and clinical variables. The only subgroup suggesting a difference in BC risk was women with percent density > 50%, but this result was based on just eight subjects and two breast cancer events. These results are in contrast to women with non-proliferative disease, for whom high MBD was strongly associated with increased BC risk.

Our findings are consistent with those from a nested case–control study using women with biopsies enrolled in the Breast Cancer Detection Demonstration Project [16]. In this study of 347 BC cases and 410 age- and race-matched controls, Byrne et al. examined BC risk within categories defined by combinations of percent density assessed by Cumulus and histologic impression. For women with NP, they observed a strong dose–response association with density: ORs = 1.0 (ref) for women with <50% density, 2.5 for PD of 50-74%, and 5.8 for PD ≥75%. This association attenuated for women with PDWA: ORs = 1.6 for <50%, 2.5 for 50-74%, and 3.2 for ≥75%, relative to women with NP and PD < 50%. Notably, they observed no apparent association for women with AH (ORs = 4.1 for <50%, 3.0 for 50-74%, and 2.1 for ≥75%), although they only had 99 women with AH (58 cases and 41 controls).

However, our results contrast with two other studies. Tice et al. examined BC risk with different combinations of BBD histologic impression and MBD, as measured using BI-RADS criteria, in more than 42,000 women in the Breast Cancer Surveillance Consortium (BCSC), including 2179 with AH diagnosed by community pathologists as part of a patient’s routine medical care [17]. Compared to women with non-proliferative disease and BI-RADS category 2, those with AH and BI-RADS category 4 were at the greatest increased risk of BC (*N* = 267, RR 5.34); those with AH and intermediate density were at intermediate risk [BI-RADS 2 (*N* = 768, RR 2.57) and BI-RADS 3 (*N* = 1079, RR 3.37)]; and those with AH and BI-RADS category 1 were at lowest risk (*N* = 65, RR 0.68), although confidence intervals overlapped for all AH risk estimates. The number of women with AH in this study (*N* = 2179) is considerably larger than our current study (*N* = 470), although women in our study were followed for a longer period of time (median 13.5 years compared to 6.1). When we limited our study to the first ten years of follow-up, we found similar null associations compared to our overall results, albeit with lower precision of estimates.

Reimers et al. examined BC risk associations in 815 women at high risk of breast cancer, with available histologic impression and with MBD data measured used the BI-RADS criteria [18]. Their study is composed of a subset of individuals enrolled in the Women at Risk Registry who had either a strong family history of breast cancer or a biopsy-proven history of LCIS or AH [24]. They reported that in the women with AH, those with BI-RADS values of 3 or 4 were at increased risk of BC (RR 4.40, 95% CI 2.24-8.67) compared to women with AH and BI-RADS of 1 or 2 (RR 1.33, 95% CI 0.54-3.26), using women with no AH and BI-RADS of 1 or 2 as the referent group. However, confidence intervals were wide and overlapped considerably between the two AH groups. The number of women in this study with AH was not reported, which makes it difficult to compare to our current study focusing only on AH. Furthermore, the average length of follow-up was 7.9 years and the number of BC events was also not specified.

Thus, of the four studies to date examining associations between MBD and BC risk in women with AH, two report suggestive but non-significant results [17, 18], while ours and Byrne et al. report decidedly null results [16]. Of note, all four studies observed overall associations between AH and BC risk, and between high MBD and BC risk, consistent with the established views. Results differed only when examining MBD and BC risk within the subset of AH individuals. Several possibilities for this discrepancy exist. First, it is possible that sample size of ours and other studies were insufficient to detect statistically significant associations. To examine this in our study, we ran a series of post-hoc power analyses based on characteristics of our cohort of 470 women. Assuming a two-sided test of hypothesis with a Type I error rate of 0.05, the observed proportions of women with low MBD and high MBD in our study, and the total observed numbers of BC events in our study, we would have 52% statistical power to detect a relative risk of 2 in high MBD women compared to low MBD women, 80% power to detect a relative risk of 2.6, and greater than 90% power to detect relative risks of 3 or larger. Thus, we have a sufficient sample size to pick up large differences in BC risk similar to those found in previous non-AH studies [13, 14], but modest sample size to pick up small or intermediate differences.

Another possible explanation for the lack of association is that women with AH and/or high MBD may have been selectively prescribed chemopreventive SERMs such as tamoxifen or raloxifene to reduce their risk of BC, which in turn could have altered any observed associations between MBD and BC risk. Among the 470 women in our study, at least 20 had documented evidence of being prescribed tamoxifen or raloxifene subsequent to initial biopsy and (for the 3 of 20 who developed BC) at least six months prior to BC diagnosis. We ran sensitivity analyses excluding these women and still found no evidence of an association between MBD and BC risk (SIR = 3.51 for low MBD, 3.47 for moderate MBC, 3.33 for high MBD, test for heterogeneity *p* = 0.98). None of the three other studies mentioned prevalence of use of chemopreventive agents in their findings. However, given the fact that clinical information was collected prior to 1990 for Byrne et al. and prior to 2006 for Reimers et al., before tamoxifen and raloxifene were commonly used preventively, it is unlikely that these agents affected risk associations for those studies.

A biologically viable explanation is that high MBD promotes the development of precancerous lesions such as AH, which in turn are associated with increased BC risk. Perhaps high MBD provides a permissive microenvironment for epithelial abnormalities to progress to pre-malignancy, but once a woman progresses to AH the density in the microenvironment has no further promoting effect. MBD is composed of both epithelial and stromal components. It is possible that the BC risk associated with AH reflects the risk related to the epithelial component of MBD. It is also believed that stromal growth factors may influence the epithelium, resulting in abnormalities such as AH which in turn influences subsequent BC risk [25]. If this was the case, one would expect to see a strong positive association between MBD and presence of AH. This indeed has been reported by several studies, including the current one. Boyd and colleagues found that women with high MBD had a 9.7-fold increased risk of developing AH and/or DCIS compared to those with low MBD [15]. Cuzick et al. found that women with a personal history of AH were 20 times more likely to have high PD (defined as ≥50%) than those with no previous breast biopsy, and 12 times more likely to have high PD than those with non-proliferative disease [26]. Our finding that women with AH were more than twice as likely to have high MBD as those with NP corroborates these results.

Although the vast majority of our results were null, we did observe a possible increased risk in BC for women with AH and PD > 50% (SIR 4.98, 95% CI 0.60-17.92). However, this result did not approach statistical significance due to the small number of women with this phenotype and so needs to be verified in an external cohort.

An interesting finding from this study was that women with PP MBD measures were more likely to fall into the high and low MBD categories than those with BI-RADS measures, who tended to cluster in the moderate category. This may indicate that PP is better at stratifying levels of MBD than BI-RADS. The PP does attempt to assess density amount/proportion and patterns (i.e. nodular vs. diffuse), while the BI-RADS density historically emphasized proportions. Regardless, associations of MBD with BC risk were similar in the PP and BI-RADS subsets of women.

Our study has several notable strengths. AH for each study participant was confirmed by a single breast pathologist with broad breast research experience. This is an important consideration given the known misclassification issues for these lesions [27]. Detailed information on clinical and demographic attributes, and post-biopsy follow-up for cancer events, was ascertained based on questionnaires and review of Mayo Clinic’s unified medical record and tumor registry database. It should be noted that study participants were primarily Caucasian, and all were seen at the same institution in the Upper Midwest, so geographic and racial/ethnic makeup of the cohort is somewhat homogeneous. The PP and BI-RADS MBD measures used in our primary analyses are subjective but clinically relevant and have been consistently associated with BC risk [12, 28–38] including in our own populations [39–41]. We examined multiple measures of breast density, including PP, BI-RADS and PD. Moreover, Byrne et al. [16] found similar results to ours using PD measures. Finally, some of the subset analyses resulted in small cell sizes, making it difficult to state unequivocally that there is no association across all subgroups.

## Conclusion

In summary, we evaluated the impact of mammographic density on breast cancer risk in women with AH, based within a cohort of women with benign breast disease. Women with AH were more likely to have higher mammographic density than women without AH. Although mammographic density was associated with higher risk in women without AH, it did not stratify risk in women with AH. Therefore, our results suggest that MBD measures may not play as important a role when making management decisions for women with AH than for women with other forms of benign breast disease

## Additional files

Additional file 1: Summary statistics of eligible women. (DOCX 18 kb)
Additional file 2: Associations of MBD measurement type with demographic and clinical variables in women with atypical hyperplasia. (DOCX 18 kb)
Additional file 3: Associations of parenchymal pattern (PP) and BI-RADS MBD measures with breast cancer risk in women with atypical hyperplasia, using the original four-level categorization. (DOCX 16 kb)
Additional file 4: Associations of extent of mammographic breast density with breast cancer risk in women with atypical hyperplasia using Cox proportional hazards regression analysis. (DOCX 17 kb)

## Abbreviations

ADH: Atypical ductal hyperplasia
AH: Atypical hyperplasia
AI: Aromatase inhibitors
ALH: Atypical lobular hyperplasia
BBD: Benign breast disease
BC: Breast cancer
BI-RADS: Breast imaging reporting and data system
CI: Confidence intervals
DCIS: Ductal carcinoma
LCIS: Lobular carcinoma in situ
LI: Lobular involution
MBD: Mammographic breast density
NP: Nonproliferative disease
PD: Percent data
PDWA: Proliferative disease without atypia
PP: Parenchymal pattern
SEER: Surveillance epidemiology and end results
SERMs: Selective estrogen receptor modulator
SIRs: Standardized incidence ratios
