# Supplementary material for: Mammographic breast density and risk of breast cancer in women with atypical hyperplasia: an observational cohort study from the Mayo Clinic Benign Breast Disease (BBD) cohort
Source: BMC Cancer. 2017 Jan 31;17:84. doi: 10.1186/s12885-017-3082-2 (PMC5282712; doi:10.1186/s12885-017-3082-2)
Supplement: Additional file 1: — Summary statistics of eligible women. (DOCX 18 kb) [file 12885_2017_3082_MOESM1_ESM.docx]

Additional File 1. Summary statistics of eligible women.

| Histologic impression | Age at biopsy | MBD value^1^ | No.women | BMI median (range) | No. breast cancer events |
| --- | --- | --- | --- | --- | --- |
| **NP** | **< 45** | **1** | 169 | 29 (24-34) | 5 |
|  |  | **2** | 147 | 29 (25-34) | 5 |
|  |  | **3** | 269 | 26 (23-30) | 20 |
|  |  | **4** | 658 | 23 (21-27) | 50 |
|  | **45-55** | **1** | 159 | 29 (25-34) | 8 |
|  |  | **2** | 172 | 28 (24-33) | 7 |
|  |  | **3** | 242 | 27 (23-31) | 24 |
|  |  | **4** | 322 | 25 (23-28) | 25 |
|  | **> 55** | **1** | 371 | 28 (24-31) | 17 |
|  |  | **2** | 352 | 27 (24-31) | 19 |
|  |  | **3** | 404 | 26 (23-29) | 23 |
|  |  | **4** | 267 | 24 (22-27) | 20 |
| **PDWA** | **< 45** | **1** | 44 | 29 (25-35) | 4 |
|  |  | **2** | 43 | 29 (24-35) | 0 |
|  |  | **3** | 123 | 25 (23-29) | 10 |
|  |  | **4** | 276 | 23 (21-27) | 17 |
|  | **45-55** | **1** | 93 | 28 (24-33) | 6 |
|  |  | **2** | 109 | 29 (25-34) | 9 |
|  |  | **3** | 208 | 26 (23-31) | 22 |
|  |  | **4** | 271 | 24 (22-28) | 40 |
|  | **> 55** | **1** | 227 | 29 (25-33) | 21 |
|  |  | **2** | 265 | 28 (24-32) | 21 |
|  |  | **3** | 356 | 26 (24-30) | 51 |
|  |  | **4** | 254 | 26 (22-29) | 21 |
| **AH** | **< 45** | **1** | 7 | 32 (26-34) | 2 |
|  |  | **2** | 4 | 32 (27-34) | 1 |
|  |  | **3** | 11 | 26 (22-30) | 1 |
|  |  | **4** | 32 | 24 (21-26) | 4 |
|  | **45-55** | **1** | 9 | 27 (24-33) | 0 |
|  |  | **2** | 27 | 28 (24-32) | 6 |
|  |  | **3** | 49 | 28 (25-31) | 9 |
|  |  | **4** | 67 | 24 (22-28) | 14 |
|  | **> 55** | **1** | 53 | 27 (24-32) | 10 |
|  |  | **2** | 56 | 29 (25-32) | 9 |
|  |  | **3** | 84 | 27 (23-30) | 15 |
|  |  | **4** | 71 | 25 (22-29) | 11 |

NP, non-proliferative disease; MBD, mammographic brease density.

1. 1=parenchymal pattern N1 or BI-RADS low density; 2=parenchymal pattern P1 or BI-RADS average density; 3=parenchymal pattern P2 or BI-RADS high density; 4=parenchymal patter DY or BI-RADS very high density.
