# Supplementary material for: Mammographic breast density and risk of breast cancer in women with atypical hyperplasia: an observational cohort study from the Mayo Clinic Benign Breast Disease (BBD) cohort
Source: BMC Cancer. 2017 Jan 31;17:84. doi: 10.1186/s12885-017-3082-2 (PMC5282712; doi:10.1186/s12885-017-3082-2)
Supplement: Additional file 2: — Associations of MBD measurement type with demographic and clinical variables in women with atypical hyperplasia. (DOCX 18 kb) [file 12885_2017_3082_MOESM2_ESM.docx]

Additional File 2. Associations of MBD measurement type with demographic and clinical variables in women with atypical hyperplasia.

|  | Type of density measure | | |  |
| --- | --- | --- | --- | --- |
|  | BI-RADS (N=188) | PP (N=282) | Total (N=470) | p-value^1^ |
| **Age of BBD** |  |  |  | 0.72 |
| <45 | 19 (10.1%) | 35 (12.4%) | 54 (11.5%) |  |
| 45-55 | 63 (33.5%) | 89 (31.6%) | 152 (32.3%) |  |
| 55+ | 106 (56.4%) | 158 (56.0%) | 264 (56.2%) |  |
|  |  |  |  |  |
| **Number of Atypical Foci** |  |  |  | 0.58 |
| 1 | 107 (56.9%) | 162 (57.4%) | 269 (57.2%) |  |
| 2 | 44 (23.4%) | 74 (26.2%) | 118 (25.1%) |  |
| 3+ | 37 (19.7%) | 46 (16.3%) | 83 (17.7%) |  |
| **Type of Atypia** |  |  |  | 0.04 |
| ADH | 100 (53.2%) | 122 (43.3%) | 222 (47.2%) |  |
| ALH | 74 (39.4%) | 145 (51.4%) | 219 (46.6%) |  |
| ADH and ALH | 14 (7.4%) | 15 (5.3%) | 29 (6.2%) |  |
|  |  |  |  |  |
| **BMI at Biopsy** |  |  |  | 0.16 |
| Missing | 2 | 3 | 5 |  |
| <25 | 76 (40.9%) | 128 (45.9%) | 204 (43.9%) |  |
| 25-29 | 46 (24.7%) | 78 (28.0%) | 124 (26.7%) |  |
| 30+ | 64 (34.4%) | 73 (26.2%) | 137 (29.5%) |  |
|  |  |  |  |  |

1. Chi-square test
