# Supplementary material for: Mammographic breast density and risk of breast cancer in women with atypical hyperplasia: an observational cohort study from the Mayo Clinic Benign Breast Disease (BBD) cohort
Source: BMC Cancer. 2017 Jan 31;17:84. doi: 10.1186/s12885-017-3082-2 (PMC5282712; doi:10.1186/s12885-017-3082-2)
Supplement: Additional file 3: — Associations of parenchymal pattern (PP) and BI-RADS MBD measures with breast cancer risk in women with atypical hyperplasia, using the original four-level categorization. (DOCX 16 kb) [file 12885_2017_3082_MOESM3_ESM.docx]

Additional File 3. Associations of parenchymal pattern (PP) and BI-RADS MBD measures with breast cancer risk in women with atypical hyperplasia, using the original four-level categorization.

| Characteristic | No. Women | Observed Events | Expected Events | SIR (95% CI) | p-value^1^ |
| --- | --- | --- | --- | --- | --- |
| **PP** |  |  |  |  | 0.97 |
| N1 | 60 | 11 | 3.16 | 3.48 (1.74, 6.23) |  |
| P1 | 32 | 7 | 1.93 | 3.62 (1.46, 7.45) |  |
| P2 | 59 | 10 | 3.46 | 2.89 (1.39, 5.32) |  |
| DY | 131 | 24 | 7.33 | 3.28 (2.10-4.87) |  |
|  |  |  |  |  |  |
| **BI-RADS** |  |  |  |  | 0.96 |
| Fatty | 9 | 1 | 0.37 | 2.73 (0.07, 15.13) |  |
| Scattered | 55 | 9 | 2.58 | 3.49 (1.60, 6.64) |  |
| Heterogeneously Dense | 85 | 15 | 3.80 | 3.95 (2.21, 6.51) |  |
| Extremely Dense | 39 | 5 | 1.59 | 3.14 (1.02, 7.31) |  |

Standardized incidence ratios and corresponding 95% confidence intervals, comparing the observed number of breast cancer events to those expected based on incidence rates from Iowa SEER data. Analyses account for the effects of age and calendar period.

1. P-value, test of heterogeneity in SIRs.
