# Supplementary material for: Mammographic breast density and risk of breast cancer in women with atypical hyperplasia: an observational cohort study from the Mayo Clinic Benign Breast Disease (BBD) cohort
Source: BMC Cancer. 2017 Jan 31;17:84. doi: 10.1186/s12885-017-3082-2 (PMC5282712; doi:10.1186/s12885-017-3082-2)
Supplement: Additional file 4: — Associations of extent of mammographic breast density with breast cancer risk in women with atypical hyperplasia using Cox proportional hazards regression analysis. (DOCX 17 kb) [file 12885_2017_3082_MOESM4_ESM.docx]

Additional File 4. Associations of extent of mammographic breast density with breast cancer risk in women with atypical hyperplasia using Cox proportional hazards regression analysis.

| Characteristic | No. Unaffected | No. Affected | Hazard Ratio (95% CI)^1^ | p-value^1^ | Hazard Ratio (95% CI)^2^ | p-value^2^ |
| --- | --- | --- | --- | --- | --- | --- |
| **Breast Density** |  |  |  | 0.78 |  | 0.69 |
| Low | 57 (14.7%) | 12 (14.6%) | 1.00 (ref) |  | 1.00 (ref) |  |
| Moderate | 190 (49.0%) | 41 (50.0%) | 0.98 (0.52, 1.87) |  | 0.90 (0.44, 1.81) |  |
| High | 141 (36.3%) | 29 (35.4%) | 0.84 (0.43, 1.65) |  | 0.75 (0.37, 1.53) |  |
|  |  |  |  |  |  |  |
| **Percent Density** |  |  |  | 0.76 |  | 0.47 |
| 0-10% | 51 (28.3%) | 8 (25.0%) | 1.00 (ref) |  | 1.00 (ref) |  |
| 11-25% | 57 (31.7%) | 12 (37.5%) | 1.40 (0.57, 3.42) |  | 1.76 (0.68, 4.60) |  |
| 26-50% | 66 (36.7%) | 10 (31.3%) | 0.99 (0.39, 2.52) |  | 1.25 (0.42, 3.72) |  |
| 51+% | 6 (3.3%) | 2 (6.3%) | 1.74 (0.37, 8.22) |  | 3.04 (0.56, 16.47) |  |

1. Adjusted for age at biopsy and body mass index.
2. Adjusted for age at biopsy, body mass index, type of MBD measure (BI-RADS vs. PP) and extent of lobular involution.
